# A Facile Electrochemical Sensor for Nonylphenol Determination Based on the Enhancement Effect of Cetyltrimethylammonium Bromide

**DOI:** 10.3390/s130100758

**Published:** 2013-01-07

**Authors:** Qing Lu, Weina Zhang, Zhihui Wang, Guangxia Yu, Yuan Yuan, Yikai Zhou

**Affiliations:** MOE Key Lab of Environment and Health, Institute of Environmental Medicine, School of Public Health, Tongji Medical College, Huazhong University of Science and Technology, Wuhan 430030, China; E-Mails: qi_weiliao@126.com (Q.L.); nanazhang2012@126.com (W.Z.); wangzhihui02003@yahoo.com.cn (Z.W.); yu266021@163.com (G.Y.); yuan639@126.com (Y.Y.)

**Keywords:** nonylphenol (NP), cetyltrimethylammonium bromide (CTAB), determination, electrochemical sensor

## Abstract

A facile electrochemical sensor for the determination of nonylphenol (NP) was fabricated in this work. Cetyltrimethylammonium bromide (CTAB), which formed a bilayer on the surface of the carbon paste (CP) electrode, displayed a remarkable enhancement effect for the electrochemical oxidation of NP. Moreover, the oxidation peak current of NP at the CTAB/CP electrode demonstrated a linear relationship with NP concentration, which could be applied in the direct determination of NP. Some experimental parameters were investigated, such as external solution pH, mode and time of accumulation, concentration and modification time of CTAB and so on. Under optimized conditions, a wide linear range from 1.0 × 10^−7^ mol·L^−1^ to 2.5 × 10^−5^ mol·L^−1^ was obtained for the sensor, with a low limit of detection at 1.0 × 10^−8^ mol·L^−1^. Several distinguishing advantages of the as-prepared sensor, including facile fabrication, easy operation, low cost and so on, suggest a great potential for its practical applications.

## 1. Introduction

Nonylphenol (NP) is a degradation product of alkylphenol ethoxylates [1,2], which has been widely used in industry, business or household as detergents, emulsifiers, wetting agents, and dispersing agents [3,4] for more than 40 years. As a result of its extensive usage, NP can enter the environment and the human body via three ways. First, NP is discharged into the environment directly as industrial and agricultural waste. Due to its chemical properties, NP can be adsorbed onto and accumulated in river sediments and then enter the human body through the water supply system [5]. Second, NP can enter processed foods directly from food packages. The major source of NP residues in food package is the oxidation of trisnonylphenylphosphite (TNPP), which is usually utilized as an antioxidant in polymeric materials, especially in PVC [6]. Last, NP can enter the human body directly through the food chain. NP can be accumulated in the organs of fish and birds, and then enter human body through food chain because of its high stability and lipid solubility [7]. Compared to alkylphenol ethoxylates, NP is much more toxic and persistent in the environment [8]. The risk of NP has attracted more and more attention recently. On the one hand, some studies show that NP can induce cell death by inhibiting the activity of endoplasmic reticulum Ca^2+^ pumps and produce oxidative stress by enhancing reactive oxygen species (ROS) [7]. On the other hand, it is found that NP has estrogenic activity and is therefore termed a xenoestrogen or endocrine disrupting chemical (EDC) [9,10]. EDCs are environmental contaminants that can interfere with endocrine glands and their hormones or where the hormones act, the target tissues [11]. Their potential adverse effects are able to induce, in males of many aquatic and semi-aquatic species, the synthesis of vitellogenin (VTG) [12] which will lead to feminization and imposex in several aquatic organisms [13,14].

Considering that NP can cause huge potential harm to human health and environment as described above, it is of great significance to develop sensitive methods for the detection of NP. To date, commonly used methods for NP detection include GC-MS [15], immunosensors [16], HPLC [17] and LC-ESI-MS [18]. Although these conventional methods are reliably sensitive, shortcomings such as high-cost and being time-consuming greatly restrict their applications. Electrochemical sensors have some virtues including high sensitivity, rapid response and low consumption, which have been successfully applied to the detection of NP. For instance, an electrochemical sensor based on titanium oxide and gold nanoparticles has been developed for the determination of NP [19]. However, the reported electrochemical sensors had some limitations, such as requiring a complex fabrication process, involving mediators or probes, and being based on enzyme catalysis, which are unfavorable for practical utilization and large scale manufacture. Thus, the development of simple facile and novel electrochemical sensors for NP determination is still a challenge.

Cetyltrimethylammonium bromide (CTAB) is a kind of cationic surfactant which has been extensively employed to enhance the sensitivity of electrochemical sensors [20]. It has a hydrophilic head on one side and a long hydrophobic tail on the other side. Thus, CTAB can not only endow the electrode/solution interface with different electrical properties, but also be adsorbed to the electrode surface or be aggregated into supermolecular structures to change the electrochemical process [21]. In this work, a facile electrochemical sensor using the enhancement effect of CTAB was developed for the detection of NP. Since CTAB could form a bilayer on the surface of the CP electrode via hydrophobic interactions between the hydrophobic long chain of CTAB molecules and the paraffin oil in the carbon paste, a remarkable enhancement of the electrochemical oxidation of NP was achieved. The sensor also presented a rapid and sensitive amperometric response to NP, which makes it great potential for NP detection.

## 2. Experimental Section

### 2.1. Reagents and Chemicals

Cetyltriethylammnonium bromide (CTAB, purchased from Sinopharm Chemical Reagent Co. Ltd. (Shanghai, China) was dissolved in water to prepare 0.01 mol·L^−1^ stock solution. Before use, the stock solution was treated in an ultrasonic bath for 5 minutes to ensure homogeneity. The stock solution of 0.1 mol·L^−1^ nonylphenol (NP, purchased from Aladdin Chemistry Co. Ltd., Shanghai, China) was prepared with anhydrous ethanol and kept in a refrigerator at 4 °C. Phosphate buffered solution (PBS, 0.1 mol·L^−1^, pH 7.0) was used as electrolyte and stored at room temperature. Graphite powder (chemically pure grade) and paraffin oil (chemically pure grade) were purchased from Shanghai Reagent Co. Ltd., (Shanghai, China) and Tianjin Reagent Co. Ltd., (Tianjin, China), respectively. Other chemicals were analytical reagents and used without any further purification. Triple distilled water was used in experiments.

### 2.2. Preparation of the Modified Electrode

The carbon paste (CP) working electrode was prepared as reported in the literature [22]: briefly, 100 mg graphite powder and 16 μL paraffin oil were mixed to produce a homogenous carbon paste. The carbon paste was then packed into the cavity of a homemade electrode and polished on a weighing paper to make a smooth surface. Then, 10 μL of CTAB solution with different concentrations was pipetted onto the surface of the smoothed CP electrode. Five minutes later, the electrode was rinsed with triple distilled water to remove the unadsorbed modifier and dried in air.

### 2.3. Instruments

Electrochemical experiments were performed with a CHI660C electrochemical workstation (CH Instruments, Shanghai, China). A conventional three-electrode system, including a working CTAB/CP electrode, a platinum wire counter electrode and a saturated calomel reference electrode (SCE), was employed. All experiments were carried out at room temperature (25 °C).

### 2.4. Preparation of Real Sample

A PVC mineral water bottle was purchased from a local supermarket. The bottle was cut into pieces with scissors and washed twice with triple distilled water. Then a real sample was prepared by the following process as previously reported [23]. The plastic bottle pieces (1.00 g) were added to a beaker containing 30 mL of triple distilled water, and then the beaker was sealed with Parafilm. Next, the beaker was ultrasonicated for 30 min and then immersed in a water bath at 70 °C for 48 h. And then, the liquid phase in the beaker was collected by filtration. Finally, the obtained liquid was rotary evaporated and then dissolved in anhydrous ethanol (2 mL). The spiked sample solution was prepared by adding a known amount of NP standard solution into the final obtained liquid.

## 3. Results and Discussion

### 3.1. Cyclic Voltammetric Behavior of NP

**Figure 1.**
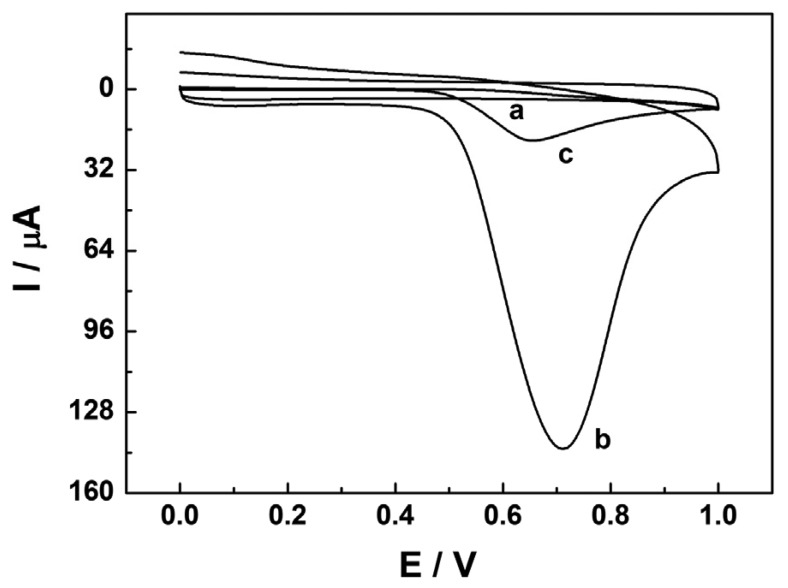
Cyclic voltammograms at 100 mV/s in 0.1 M PBS (pH 7.0) for the CTAB/CP electrode (a) in the absence of NP and (b) in the presence of 20 μM NP, and (c) the bare CP electrode in the presence of 20 μM NP.

In order to study the unique properties and promising potential of the proposed sensor for NP determination, the electrochemical behavior of NP at the CTAB/CP electrode was examined by using cyclic voltammetry (CV) in pH 7.0 PBS (0.1 mol·L^−1^), as shown in Figure 1. Obviously, no peak is observed at the CP electrode modified with 7.0 × 10^−4^ mol·L^−1^ CTAB (curve a) in the absence of NP. A weak anodic peak located at 0.655 V at the bare CP electrode is obtained (curve c) in the presence of NP. Contrasted well with the CP electrode, the CP electrode modified with 7.0 × 10^−4^ mol·L^−1^ CTAB gives a much bigger oxidation peak in the presence of NP with the same concentration (curve b), indicating that the presence of CTAB can enhance the oxidation of NP at the CP electrode. It has been confirmed in our previous work [22] that CTAB could be firmly adsorbed on the CP electrode surface via hydrophobic interactions between the hydrophobic long chain of CTAB molecules and the paraffin oil in the carbon paste. A bilayer of CTAB was formed on the CP electrode when the concentration of CTAB was 7.0 × 10^−4^ mol·L^−1^ [20]. The formed CTAB bilayer was hydrophobic, which would accumulate NP via hydrophobic interactions and increase NP's concentration on the electrode surface and then accelerate the electron transfer between NP and the CP electrode. No apparent reduction peaks are observed in the reverse scan at the CP electrode and the CTAB/CP electrode (curves c and b), suggesting that the oxidation of NP at the electrode is a totally irreversible electrochemical process.

The effect of the scan rate (*ν*) on the electrochemical behavior of NP at the CTAB/CP electrode was examined by CV. The oxidation peak current of NP at the modified electrode was increasing with the increase of the scan rate. In the range of 60–420 mV/s, the peak current (*i*_p_) had a linear relationship with the square root of the scan rate, suggesting the oxidation of NP at the CTAB/CP electrode was a diffusion-controlled reaction. Meanwhile, the oxidation peak potential (E_pa_) of NP at the CTAB/CP electrode shifted positively with the increase of the scan rate. For a totally irreversible diffusion-controlled electrode process, E_pa_ can be defined by the following Equation [24]:

$$E_{pa}=E_{0}+\left(\frac{RT}{\alpha nF}\right)\ln\left(\frac{{\text{RTk}}^{0}}{\alpha nF}\right)+\left(\frac{RT}{\alpha nF}\right)\ln\nu$$

where *α* is the anodic transfer coefficient, *k*° is the standard rate constant of the reaction, *n* is the number of transferred electrons, *ν* is the scan rate, and *E*_0_ is the formal redox potential. Other symbols have their usual meanings. Therefore, the value of *αn* was calculated to be about 0.62. Generally, for a totally irreversible electrode process, the value of *α* is between 0.3 and 0.7 [24]. It has been discovered that one or two transferred electrons are involved in the direct oxidation of phenolic compounds to generate phenoxy radical and quinone, respectively [25]. However, the oxidation of NP is complicated because its oxidation was found to start from the degradation of linear alkyl chains, instead of the phenol ring [26]. Thus, in this work, for the oxidation of NP at the CTAB/CP electrode, the number of transferred electrons is estimated to about 1–2, which is consistent with a previous report [25]. The influence of the external solution pH on the oxidation potential of NP at the CTAB/CP electrode was also investigated by CV. When the pH value increased from 4.0 to 10.0, the oxidation potential of NP shifted negatively. Moreover, the potential shift displayed a linear relationship with the increase of the pH value. It suggested the oxidation of NP at the CTAB/CP electrode was a proton-transfer involved reaction. A slope of E *vs.* pH was obtained to be about −69 mV/pH, indicating an equal number of protons and electrons were transferred.

### 3.2. Optimization of NP Detection at the CTAB/CP Electrode

In order to improve the sensitivity of the NP signal detected with the proposed sensor, some experimental parameters, such as external solution pH, mode and time of accumulation, concentration and modification time of the modifier (CTAB) and so on, were investigated and optimized.

**Figure 2.**
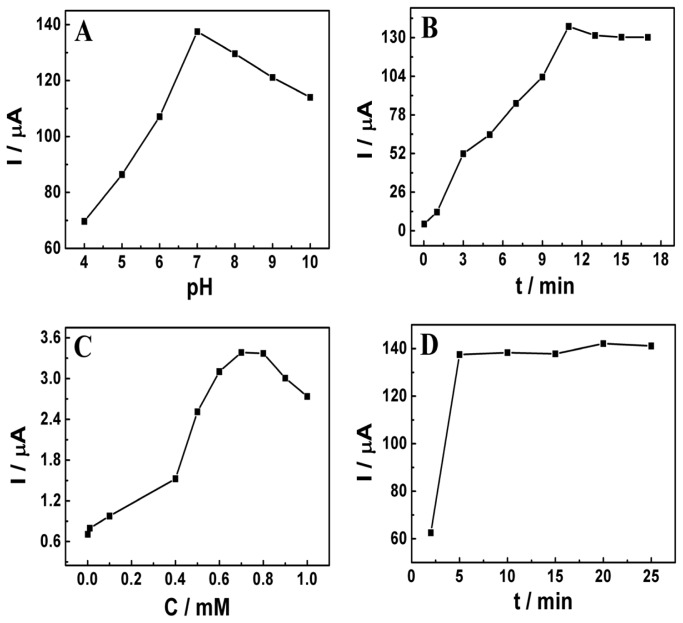
(**A**) Relationship between peak current and external solution pH; (**B**) Effect of accumulation time on the oxidation peak current of 20 μM NP; (**C**) Influence of CTAB concentration on the oxidation peak current of 20 μM NP; (**D**) Effect of modification time on the oxidation peak current of 20 μM NP.

The influence of the external solution pH on the oxidation of NP at the CTAB/CP electrode was studied by CV in the range from 4.0 to 10.0. As illustrated in Figure 2(A), it is found that the oxidation peak current of NP at the modified electrode increases gradually with the increase of pH from 4.0 to 7.0, and then declines with further increase. As mentioned above, the oxidation of NP at the CTAB/CP electrode is a proton-transfer involved reaction. Thus, the pH of electrolyte may have effects on the rate of electrochemical reaction. Here, a neutral pH is much better for the oxidation of NP. Otherwise, in acid or basic media, the current decreases. Therefore, a pH value of 7.0 was chosen as the optimized external solution pH for the subsequent analytical experiments.

In general, an accumulation procedure can improve the sensitivity of electrochemical sensors since it can increase the concentration of analyte on the electrode surface. Two key accumulation parameters, mode and time, were investigated in this work, respectively. For the mode of accumulation, open circuit mode and closed circuit mode were examined. No significant difference was observed between those two modes. Considering the simplicity of operation, closed circuit mode was chosen for the following experiments. The influence of the accumulation time on the electrochemical response of NP at the modified electrode is also studied, as shown in Figure 2(B). Obviously, the oxidation peak current of NP increases gradually with the increase of the accumulation time from 0 to 11 min, and then tends to be stable with further increase of the accumulation time. The stable electrochemical responses may have relationship with the saturated adsorption of NP at the modified electrode. Thus, an optimized accumulation time of 11 min was employed in further experiments.

It was found that the presence of CTAB could enhance the oxidation of NP at the CP electrode. As the electrode modifier, the concentration of CTAB on the surface of the CP electrode would have a great effect on the electrochemical response of NP. During the process of electrode modification, the concentration of CTAB on the surface of the electrode was determined by the concentration and the modification time of the pipetted modifier, which were also optimized. Figure 2(C) displays the effect of the pipetted CTAB concentration on the response of NP at the modified electrode. Obviously, the oxidation peak current of NP increases when the CTAB concentration is changing from 1.0 × 10^−6^ mol·L^−1^ to 7.0 × 10^−4^ mol·L^−1^. When the concentration exceeds 7.0 × 10^−4^ mol·L^−1^, the peak current drops down gradually. In our previous works [22], it was found that the pipetted CTAB concentration affected the existing molecules' status in solution and then the molecules' arrangement on the electrode/solution interface, leading to the influences on the electrochemical response of NP. There were three types of adsorptive behavior for CTAB adsorbed on the surface of the CP electrode, monomer adsorption, monolayer adsorption and bilayer adsorption [20]. At a lower concentration (1.0 × 10^−6^ mol·L^−1^), monomer adsorption was prevailing. With the increasing of concentration (from 1.0 × 10^−6^ mol·L^−1^ to 4.0 × 10^−4^ mol·L^−1^), the adsorptive behavior of CTAB was changing from monomer adsorption to monolayer adsorption, and then became bilayer adsorption (from 4.0 × 10^−4^ mol·L^−1^ to 7.0 × 10^−4^ mol·L^−1^) [20]. In the cases of monomer adsorption and monolayer adsorption, the modified electrode was partially positive-charged and hydrophilic, which could not accumulate NP. However, for CTAB bilayer, NP could be accumulated to the modified electrode via hydrophobic interactions. Therefore, a pipetted CTAB concentration of 7.0 × 10^−4^ mol·L^−1^ was chosen as the optimized value. The influence of the modification time on the electrochemical response of NP was also investigated and illustrated in Figure 2(D). The oxidation peak current of NP increases visibly with the increase of the modification time from 2 min to 5 min. However, when the time is longer than 5 min, the peak current reaches a stable value. The phenomena may also relate to CTAB's adsorptive behaviors. At the beginning of adsorption, monomer adsorption was prevailing. When the time was increasing, monomer adsorption was changing to monolayer adsorption, and then bilayer adsorption. Moreover, once a CTAB bilayer was completely formed on the surface of the CP electrode, the adsorption/desorption rate of CTAB became very slow [20]. Therefore, before the formation of CTAB bilayer, the surface of the modified electrode was defective and partially hydrophilic, leading to the unstable oxidation peak current. After the complete formation of CTAB bilayer, the modified electrode surface was steady and hydrophobic, resulting in little increase in the peak current. Consequently, a modification time of 5 min was chosen.

### 3.3. Detection of NP at the CTAB/CP Electrode

**Figure 3.**
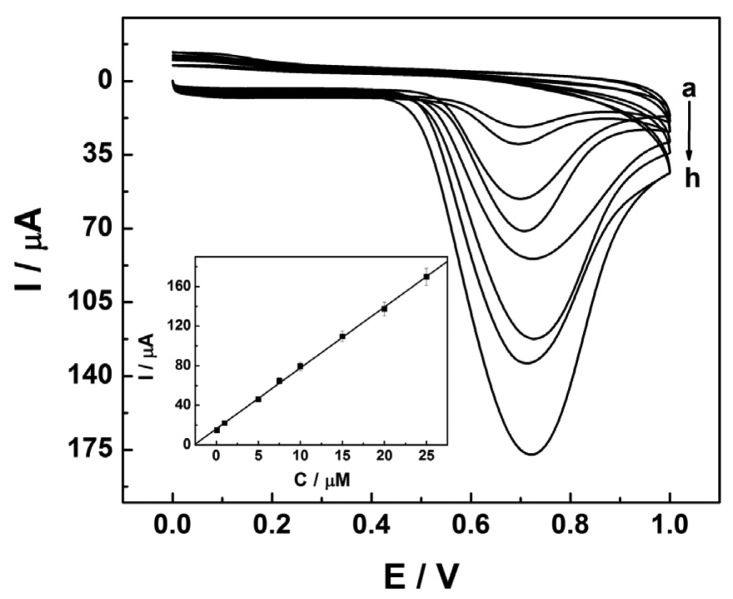
Cyclic voltammograms at the CTAB/CP electrode in PBS (pH 7.0) containing (a–h): 0.1, 1, 5, 7.5, 10, 15, 20, 25 μM NP. The inset is a plot of the linear relationship between the oxidation peak current and the concentration of NP.

Under the above optimized experiment parameters, CV was utilized to detect NP of different concentrations at the CATB/CP electrode. As displayed in Figure 3, an obvious increase of the oxidation peak current of NP is obtained with the increase of NP concentration. Moreover, the peak current of NP shows a linear relationship with its concentration (shown in Figure 3 inset). In the range from 1.0 × 10^−7^ mol·L^−1^ to 2.5 × 10^−5^ mol·L^−1^, a linear regression equation is obtained as: *i* = 16.21579 + 6.15973*C* (*R* = 0.9996, *i* in μA, *C* in μM). A limit of detection as low as 1.0×10^−8^ mol·L^−1^ is estimated (S/N = 3). Compared with other electrochemical sensors, such as 3.2 × 10^−7^ mol·L^−1^ for TiO_2_-nano Au-molecularly imprinted sensor [19], 2.6 × 10^−7^ mol·L^−1^ for NiTPPS/CNTs sensor [27] and 10 μg·L^−1^ (≈4.5 × 10^−8^ mol·L^−1^) for HRP-based immunosensor [28], this is a competitive value.

### 3.4. Amperometric Response of NP at the CTAB/CP Electrode

**Figure 4.**
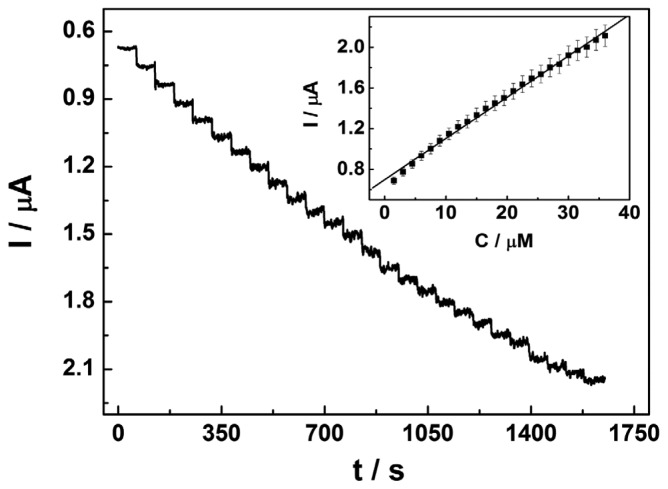
Amperometric responses at the CTAB/CP electrode at a constant potential of 0.95 V in 10 mL PBS (pH 7.0) with injection of 1.5 μM NP every 60 s. The inset is a plot of the oxidation peak current against the concentration of NP (from 1.5 to 36 μM).

Considering the sensor's potentially practical application, amperometry was performed to detect NP at the proposed sensor. For amperometry, the applied potential may have influence on the electrochemical response of the analyte. Thus, the effect of the applied potential (from 0.7 V to 1.1 V) on the current of 1.5 × 10^−6^ mol·L^−1^ NP at the modified electrode was investigated (data not shown). At 0.95 V, a maximum response was obtained. Consequently, 0.95 V was chosen as the applied potential. Figure 4 presents an amperometric I-t curve of the sensor with successive additions of 1.5 × 10^−6^ mol·L^−1^ NP (PBS, pH 7.0) at a potential of 0.95 V. Upon addition of an aliquot of NP to the electrochemical cell, the oxidation current increases steeply to reach a stable value. The sensor reaches 95% of the steady-state current in less than 2 s, indicating the oxidation reaction of NP at the sensor is fast. Moreover, the oxidation current demonstrates a linear relationship with the NP's concentration in the range from 1.5 × 10^−6^ mol·L^−1^ to 3.6 × 10^−5^ mol·L^−1^ (R = 0.998, n = 24, Figure 4 inset). The sensitivity is calculated as 0.041 μA/μM·cm^2^. The limit of detection is estimated to be 1.1 × 10^−6^ mol·L^−1^ at a signal-to-noise ratio of 3.

### 3.5. Stability, Reproducibility and Interferences of the Sensor

The stability of the sensor was investigated. When stored at 4 °C in dark for about 15 days, the sensor retained almost all its initial sensitivity to NP. The reproducibility of the sensor was estimated for a solution containing 2 × 10^−6^ mol·L^−1^ NP with the same modified electrode. The relative standard deviation (RSD) was 3.8% (n = 8). The fabrication of eight electrodes, made independently, showed an acceptable RSD reproducibility of 4.7% for the current determined at 2 × 10^−6^ mol·L^−1^ NP. Several cations, anions, surfactants and small organic molecules were investigated for their interferences with the electrochemical detection of NP by the proposed sensor. The results are listed in Table 1, indicating the sensor possesses good selectivity for NP determination.

### 3.6. Real Sample Analysis

The proposed sensor was used for real sample analysis by detection of NP in a polyvinyl chloride (PVC) mineral water bottle. The real sample was prepared following the description in Section 2.4. By the means of HPLC and the proposed sensor, no NP was found in the PVC mineral water bottle. Thus, the standard addition method was applied to investigate the performance of the proposed sensor. The results are listed in Table 2. The proposed sensor shows competitive recoveries, indicating the sensor has good accuracy and may be sufficient for practical applications.

## 4. Conclusions

In summary, a simple NP sensor was fabricated in this work. Based on the hydrophobic interactions between the hydrophobic long chain of CTAB molecules and the paraffin oil in the carbon paste, CTAB could be firmly adsorbed on the CP electrode surface and then enhance the electrochemical oxidative behavior of NP at the CP electrode. The proposed sensor demonstrated a rapid, sensitive and selective detection of NP. In view of its advantages, such as facile fabrication, easy operation, low cost and so on, the sensor shows great potential for practical applications.

## Figures and Tables

**Table 1.** Interferences of other species on 2.0 × 10^−5^ M NP.

**Interferents**	**Concentration (M)**	**Signal change (%)**
Ca^2+^	2 × 10^−3^	1.6
Cu^2+^	1 × 10^−3^	−2.7
Cl^−^	2 × 10^−3^	2.0
NO_3_^−^	2 × 10^−3^	−4.9
SDS a	1 × 10^−4^	−3.1
Spain 60	1 × 10^−4^	2.2
DTAB b	1 × 10^−4^	−2.8
Dopamine	1 × 10^−4^	−3.0
Ascorbic acid	1 × 10^−4^	2.9
Bisphenol A	1 × 10^−4^	5.2

a SDS: Sodium dodecyl sulfate;

b DTAB: dodecyltrimethylammonium bromide.

**Table 2.** Determination results of NP in PVC water bottle samples.

**Sample**	**Added (μM)**	**Found (μM)**	**Recovery (%)**
Sample 1	5	4.85	97.0
Sample 2	7.5	8.20	109.3
Sample 3	10	10.84	108.4
Sample 4	15	15.84	105.6
